# A hybrid STAMP-fuzzy DEMATEL-ISM approach for analyzing the factors influencing building collapse accidents in China

**DOI:** 10.1038/s41598-023-46778-6

**Published:** 2023-11-13

**Authors:** Xue Chen, Wanguan Qiao

**Affiliations:** 1 School of Economics and Management, China University of Mining and Technology, Xuzhou, 221116 People’s Republic of China; 2 School of General Course, Jiangsu Vocational Institute of Architectural Technology, Xuzhou, 221116 People’s Republic of China; 3 School of Economics and Management, Jiangsu Vocational Institute of Architectural Technology, Xue Yuan RD. 1, Xuzhou, 221116 Jiangsu Province People’s Republic of China

**Keywords:** Risk factors, Engineering

## Abstract

To explore the factors influencing recent construction collapse accidents, this study utilizes a sample of 355 reports on building collapse accidents from 2012 to 2022. The investigation employs the systems-theoretic accident modeling and processes (STAMP) model to retrieve 22 key causal factors of accidents from the physical, operational, managerial, and supervisory layers. Subsequently, an improved decision-making trial and evaluation laboratory (DEMATEL)-interpretive structural modeling (ISM) method is used to analyze the relationships and strengths of these influencing factors, providing a comprehensive understanding of the logical connections between the causes of building collapse accidents. The results indicate that the deep-rooted causes of building collapse accidents are primarily lax safety management at the enterprise level and the exchange of interests at the government regulatory level, which in turn affect workers at the operational level and the physical aspects of accidents on-site. Furthermore, integrating the STAMP model and the triangular fuzzy DEMATEL-ISM model overcomes the limitations of the traditional STAMP model, allowing for a more focused identification of key factors.

## Introduction

**Figure 1:**
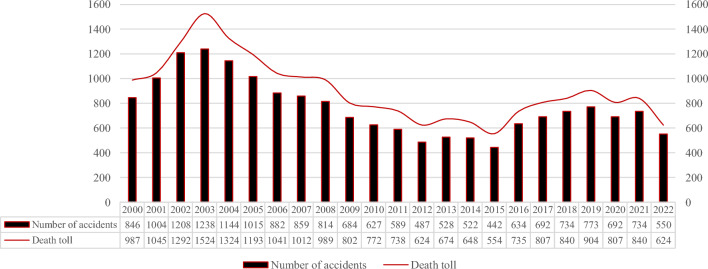
Safety accident statistics for housing and municipal engineering in China, 2000–2022. Data source: Ministry of Housing and Urban‒Rural Development of China (MHURDC).

The construction industry has consistently been one of the industrial sectors with the highest occupational safety incident rates worldwide. According to data from the Occupational Safety and Health Administration website (OSHA, 2019), approximately 20% of worker fatalities in developed countries occur in the construction industry^1^. In developing countries, this percentage rises to approximately 30–40% of occupational fatalities^2^. Reports on construction accidents in Turkey reveal that the average mortality rate in the construction sector between 2007 and 2016 was approximately 22.35 per 1000 workers, roughly four times higher than that of the manufacturing industry^3^. Malaysia has experienced a continuous and significant increase in fatal injuries since 2015, with a 30% rise in four years^4^. In China, there have been significant achievements in ensuring safety in the construction industry, with a reported death toll of 624 in 2022. The number of fatalities shows a clear downward trend, decreasing from 1208 in 2002 to 442 in 2015. However, there was a rebound in accidents and fatalities between 2015 and 2019, with a linear upward trend (Fig. 1). The safety situation of China's construction industry is still very serious.

**Figure 2:**
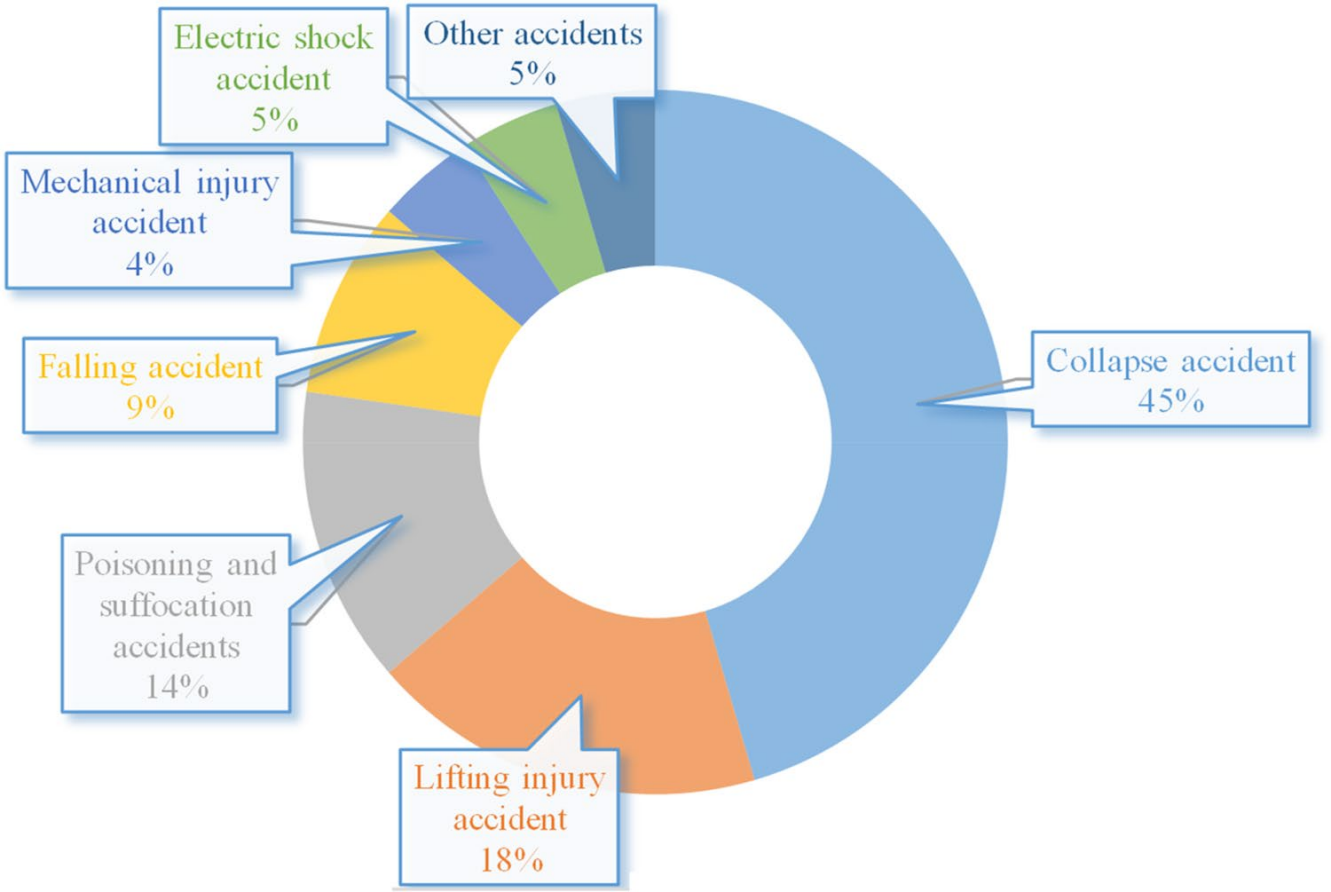
Statistics on the major housing and municipal engineering accident types in China in 2021. Data source: MHURDC.

Among construction accidents, the type with the highest frequency is falling accidents, but collapse accidents have the highest average death toll^5,6^. According to statistics from the Ministry of Housing and Urban‒Rural Development of China (MHURDC), there were 22 construction accidents with more than three deaths in 2021, including 10 collapse accidents, accounting for 45.5% of all accidents (Fig. 2). The average death rate of a collapse accident is 1.9, nearly twice the average death rate of all accidents, which is enough to see the high-risk harmfulness of collapse accidents. Once a collapse happens, it very easily causes large-scale death and injury, and the casualties are extremely heavy^7^. On June 24, 2021, at approximately 1:22 a.m., a 12-story beachfront condominium in the Miami suburb of Surfside, Florida, United States, partially collapsed, causing the deaths of 98 people. At 7:14 p.m. on March 7, 2020, a building that was part of the Jiaxin hotel collapsed in Quanzhou, Fujian Province, resulting in 29 deaths and 42 injuries, with a direct economic loss of 57.94 million yuan. The occurrence of building collapse accidents will have not only a great impact on economic development but also a negative impact on social stability^8^.

To effectively prevent building collapse accidents and ensure the safety of construction projects, it is crucial to analyze the causal factors of these accidents and to examine the interrelationships between these factors. To do so, we improve the systems-theoretic accident modeling and processes (STAMP) accident causality model based on the needs of analyzing and investigating building collapse accidents. The modified STAMP model is utilized to systematically analyze a large number of building collapse accidents through modular solidification. Subsequently, an improved decision-making trial and evaluation laboratory (DEMATEL)-interpretive structural modeling (ISM) method is employed to analyze the interrelationships between modules within the building collapse accident system. Ultimately, a hybrid model that is capable of providing a comprehensive analysis of the building collapse accident system as a whole is formed.

## Literature review

The analysis of accident influencing factors is mostly based on accident cause theory^9,10^. The development of accident cause models involves single-factor, double-factor, multifactor and complex systems^11^. A single-factor model includes the theory of accident frequency tendency proposed by Farmers and Chambers. This theory holds that an accident is caused by some personnel who are characterized by frequent accidents, and it completely denies the impact of the objective environment and equipment on the accident. The main theories of two-factor models are the accident causal chain theory proposed by Heinrich (1941) and the accident causal chain theory proposed by Byrd (1966)^12^^.^^13^. The theories of these models hold that the occurrence of accidents is influenced by not only human factors but also management factors. In multifactor models, the accident causal chain theory proposed by Surry (1969) adds institutional and social factors^14^. In recent years, popular accident cause theories based on complex systems have included the STAMP model proposed by Leveson (2003) and a nonlinear accident cause model based on the system safety-functional resonance analysis method (FRAM) proposed by Hollnagel (2012)^15,16^.

At present, the analysis of construction accidents mainly targets large-height falling accidents, and few scholars have analyzed the causes of collapse accidents. Zhang et al. (2019) used four contemporary popular accident causal models, i.e., the STAMP, AcciMap, human factors analysis and classification system (HFACS), and 2–4 models, to analyze a typical collapse accident, and they found that in accident analysis, the four models have different focuses^17^. Kale et al. (2021) analyzed 723 accidents caused by ditch collapses and pointed out that more than 50% of the collapse accidents were caused by a lack of protective measures^18^. Huang et al. (2021) discussed China's construction safety under COVID-19 and analyzed collapse accidents from five perspectives, i.e., contractors, organization and management, technical methods, participants and interactive feedback, from the perspective of the STAMP model^19^. Yan and Kim (2018) proposed a stage model of building collapse accidents and divided building collapse accidents into an incubation period, an outbreak period, a transmission period and a final stage^20^. Okunola (2021) interviewed 42 people using a random sampling method and found that the main causes of building collapse accidents in Nigeria were institutional failure and human factors^21^.

Based on the literature analysis above, many causality models applied in accident analysis tend to have their own focuses. For instance, the HFACS model primarily emphasizes the analysis of human factors, while the 2–4 model places greater emphasis on accidents caused by management failures. This difference in focus leads to a lack of comprehensiveness and detail in the analysis of accident causes^22^. The STAMP model represents the pathways of accidents using a systemic network, which aligns with the complexity of accidents. It can provide a detailed analysis of individual accidents. However, there are still two main shortcomings. First, due to the lack of a modularized classification of causal factors, its efficiency in analyzing a large number of accident statistics is not high. Second, it uncovers too many causes, making it challenging to establish the logical relationships and strengths of the mutual influences among factors. To address these limitations, we conduct a statistical analysis of a large number of building collapse accidents. The modularization of the STAMP model is solidified into four aspects: the physical, operational, managerial, and supervisory layers of building collapse accidents. This solidification enables a rapid analysis of the causes of multiple accidents. Furthermore, we incorporate the fuzzy DEMATEL-ISM method with the STAMP approach to more effectively identify the key factors contributing to accidents.

## Methodology

### STAMP

Compared with traditional analysis methods for accident cause chains, the advantage of the STAMP model lies in the fact that it fully explains the impact of interaction defects between complex system components on system safety^23^. The main reason is that the model has three basic structures: security constraints, a hierarchical safety control structure and a process model. The hierarchical safety control structure can be established based on the confirmation of hazards and safety constraints^24^. The structure has top-down characteristics. From the bottom, the safety constraints and inappropriate control behaviors of each layer in the entire hierarchical safety control structure can be identified in turn so that a dangerous defect control process can be confirmed. The interaction relationship between each control level is represented by the process model. The general process model includes a controller and control process, an output and feedback process between them, process input and output, and the interference of external information. The deficiency lies in the fact that the STAMP model emphasizes the depth and breadth of accident analysis. Therefore, the analysis requires practitioners to have very high professional background knowledge. Additionally, the analysis process is time consuming and inefficient and lacks modular cause factor classification, which is not conducive to the statistical analysis of a large number of accidents^25^. Therefore, we solidify the STAMP model and build an accident control model from four levels, physics, operation, management and supervision, to make the model more conducive to large-scale accident analysis.

### Fuzzy DEMATEL

The DEMATEL is a method of systematic factor analysis using graph theory and matrix tools^26^. Through an analysis of the logical relationship and direct influence matrix among the factors in a system, the influence degree of each factor on other factors and the affected degree can be calculated to calculate the center degree and cause degree of each factor. This method makes full use of the experience and knowledge of experts to deal with complex social problems, especially systems with uncertain relationships between factors that are more effective.

### ISM

ISM is an analytical method that explains the logical structure between influencing factors. By constructing an adjacency matrix between influencing factors, the adjacency matrix is added to the identity matrix according to Boolean operation rules. After continuous iteration, it is equal to the initial adjacency matrix and forms the final reachability matrix. The reachability set, antecedent set and common set among the influencing factors are obtained using the reachability matrix, and finally, the hierarchical structure of the system is divided.

### ***STAMP-Fuzzy ***DEMATAL***-ISM combination***

**Figure 3:**
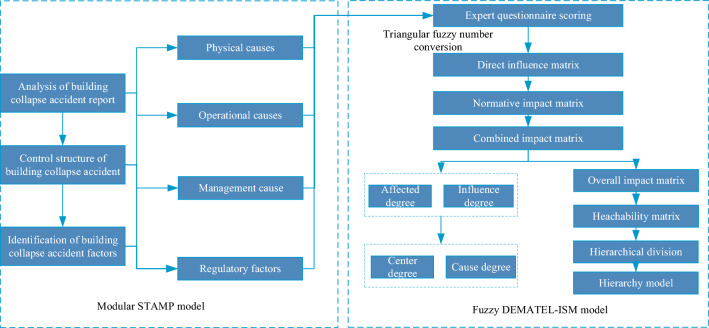
Analysis logic of a construction accident based on the STAMP-DEMATEL-ISM method.

The STAMP model is an accident cause model for complex systems that can clarify the accident causes within and between organizations from the perspective of control. However, when using the STAMP model to analyze accidents, the influence intensity of accident influencing factors cannot be obtained, which makes it impossible to grasp the key points when formulating relevant preventive measures. To overcome this defect, we introduce the DEMATEL-ISM method to analyze the interaction between factors in complex systems. However, the direct impact matrix in the DEMATEL-ISM model is usually divided into a direct relationship or no direct relationship (1 or 0), which is not suitable for accurate scoring by experts^27^. In some collapse accidents, there is no clear division between the causal factors; that is, their boundaries are ambiguous. Therefore, we introduce triangular fuzzy numbers to improve the method of obtaining the direct influence matrix in the DEMATEL-ISM model to make the obtained results more representative. The specific idea is shown in Fig. 3.

*Step 1 Identify *influencing* factors*

The STAMP method is used to determine the factors of a building collapse accident $\mathrm{S}=\left\{{S}_{1},{S}_{2},\dots ,{S}_{n}\right\}$.

*Step 2 Analyze the effects *between* indexes*

The interaction strength between indicators is difficult to directly represent using data. Therefore, this paper uses an expert scoring method to evaluate the influence degree between indicators. The expert scoring value is divided into five levels: very high (VH), high (H), low (L), very low (VL), and no effect (NE) (Table 1). Due to the subjectivity and fuzziness of expert scoring, triangular fuzzy numbers are used for transformation. Based on the correspondence between linguistic phrases and the membership function of triangular fuzzy numbers provided by Kuzu (2021), the five levels of expert scoring are transformed into triangular fuzzy numbers^28^. For the evaluation index, it is assumed that there are m experts scoring. The triangular fuzzy number corresponding to the influence degree of the *i*-th index evaluated by the* k*-th expert on the *j*-th index is $\left( {l_{ij}^{k} ,m_{ij}^{k} ,r_{ij}^{k} } \right)$.

**Table 1:** Transformation relationship of triangular fuzzy numbers.

Influence degree	Triangular fuzzy number
Very High (VH)	(0.75, 1, 1)
High (H)	(0.5, 0.75, 1)
Low (L)	(0.25, 0.5, 0.75)
Very Low (VL)	(0, 0.25, 0.5)
No Effect (NE)	(0, 0, 0.25)

*Step 3 Construct and *normalize* the direct impact matrix*

We use an improved converting fuzzy data into crisp scores (CFCS) method to convert triangular fuzzy numbers into clear numbers. The clear value of the influence degree of the *i*-th index on the *j*-th index can be obtained based on the four-stage CFCS algorithm:

(1) Standardization

1 $$ xl_{ij}^{k} = \left( {l_{ij}^{k} - \min l_{ij}^{k} } \right)/\Delta_{\min }^{\max } $$

2 $$ xm_{ij}^{k} = \left( {m_{ij}^{k} - \min l_{ij}^{k} } \right)/\Delta_{\min }^{\max } $$

3 $$ xr_{ij}^{k} = \left( {r_{ij}^{k} - \min l_{ij}^{k} } \right)/\Delta_{\min }^{\max } $$

where $$\Delta_{\min }^{\max } = \max r_{ij}^{k} - \min l_{ij}^{k}$$

(2) Calculation of the standardized values of the left-side value (*LS*) and the right-side value (*RS*)

4 $$ xls_{ij}^{k} = xm_{ij}^{k} /\left( {1 + xm_{ij}^{k} - xl_{ij}^{k} } \right) $$

5 $$ xrs_{ij}^{k} = xr_{ij}^{k} /\left( {1 + xr_{ij}^{k} - xm_{ij}^{k} } \right) $$

(3) Calculation of the total clear value after standardization

6 $$ x_{ij}^{k} = \left[ {xls_{ij}^{k} \left( {1 - xls_{ij}^{k} } \right) + xrs_{ij}^{k} xrs_{ij}^{k} } \right]/\left[ {1 - xls_{ij}^{k} + xrs_{ij}^{k} } \right] $$

7 $$ z_{ij}^{k} = \min l_{ij}^{k} + x_{ij}^{k} \Delta_{\min }^{\max } $$

(4) Normalization of the clear value

8 $$ z_{ij}^{{}} = \frac{1}{n}\left( {z_{ij}^{1} + z_{ij}^{2} + ... + z_{ij}^{n} } \right) $$

The *n* direct influence matrix is ${\text{Z}} = \left( {{\text{Z}}_{{{\text{ij}}}} } \right)_{nn}$.

The standardized parameter p is calculated and used to standardize the matrix *Z* and obtain the matrix *Q*:

9 $$p=\frac{1}{\frac{max}{1\ll i\ll n}\sum_{j=1}^{n}{Z}_{ij}} (i,j=\mathrm{1,2},\dots ,n)$$

10 $$Q=p\times Z$$

*Step 4 *Obtain* the comprehensive influence relation matrix*

11 $$ {\text{F}} = {\text{Q}}\left( {I - Q} \right)^{ - 1} $$

where I is a unit matrix.

*Step 5 Conduct *comprehensive* impact relation matrix analysis*

The influence degree (${r}_{i}$), affected degree (${c}_{j}$), center degree (${O}_{i}$) and cause degree (${P}_{i}$) of each factor are calculated.

12 $${r}_{i}=\sum_{j=1}^{n}{F}_{ij} i=\mathrm{1,2},\dots ,n$$

13 $${c}_{j}=\sum_{i=1}^{n}{F}_{ij} j=\mathrm{1,2},\dots ,n$$

14 $${O}_{i}={r}_{i}+{c}_{j}, i=j$$

15 $${P}_{i}={r}_{i}-{c}_{j}, i=j$$

*Step 6 Calculate overall *impact* relationship matrix U*

16 $$\mathrm{U}=\mathrm{I}+\mathrm{F}$$

*Step 7 Calculate *reachability* matrix K*

First, we calculate threshold *λ*. The value of *λ* is the sum of the mean and standard deviation of all elements of matrix *F*. The value rule of index *k*_*ij*_ in the *i*-th row and the *j*-th column of the reachability matrix is as follows:

17 $${k}_{ij}=\left\{\begin{array}{c}1,{ \lambda }_{ij}\ge \lambda , i,j=\mathrm{1,2},\dots ,n\\ 0{, \lambda }_{ij}<\lambda , i,j=\mathrm{1,2},\dots ,n\end{array}\right.$$

where *k*_*ij*_ = *1* indicates that factor *s*_*i*_ influences *s*_*j*_ and *k*_*ij*_ = 0 indicates that factor *s*_*i*_ has no influence on *s*_*j*_.

*Step 8 Computation of the reachability set, antecedent set, and common set*

Based on reachability matrix *K*, we calculate reachability set *R(s*_*i*_*)*, antecedent set *B(s*_*i*_*)* and common set *T(s*_*i*_*)*. The calculation rules are shown in the following formulas:

18 $$\mathrm{R}\left({s}_{i}\right)=\left\{{s}_{j}|{s}_{j}\in S,{k}_{ij}=1\right\}, i,j=\mathrm{1,2},\dots ,n$$

19 $$\mathrm{A}\left({s}_{i}\right)=\left\{{s}_{j}|{s}_{j}\in S,{k}_{ji}=1\right\}, i,j=\mathrm{1,2},\dots ,n$$

20 $$\mathrm{C}\left({s}_{i}\right)=\mathrm{ R}\left({s}_{i}\right)\cap \mathrm{A}\left({s}_{i}\right)$$

If the common set satisfies *C(s*_*i*_*)* = *R(s*_*i*_*),* the influencing factor s_i_ contained in *T(s*_*i*_*)* is the current lowest element. Then, the rows and columns corresponding to the elements contained in the bottom layer can be deleted from the reachability matrix. The steps above are repeated until all factors are stratified.

*Step 9 Draw an ISM multilevel ladder diagram based on the stratification of *influencing* factors*

## Case analysis

### Data sources

Based on the websites of the MHURDC, safety management offices, and provincial work safety supervision and administration offices as well as the portals of relevant departments searched on the internet, 355 investigation reports on building collapse accidents from 2012 to 2022 were statistically analyzed^29–31^. Each report included the basic situation of the accident, the accident process and rescue, the accident cause, an accident responsibility analysis, handling suggestions, and accident prevention and rectification measures.

From the perspective of geological factors, these accidents covered a total of 28 provincial-level administrative regions (over 80% of the geographical area of China) from 2012 to 2022. The urban distribution is concentrated in the eastern part of China, as the eastern region is more developed. From the perspective of the degree of casualties in accidents, there were 9 extremely large accidents (with a death toll of ≥ 10 people), 111 major accidents (with a death toll of ≥ 3 people), and 235 general accidents (with a death toll of < 3 people). The types of projects include both construction operations (new construction, expansion, renovation projects) and those that occur during use (maintenance, demolition, renovation, etc.). The types of collapse accidents also include typical collapse accidents such as earthwork collapses, scaffolding collapses, formwork collapses, demolition project collapses, and building collapses.

### Using the STAMP model to discover the causal factors of collapse accidents

**Figure 4:**
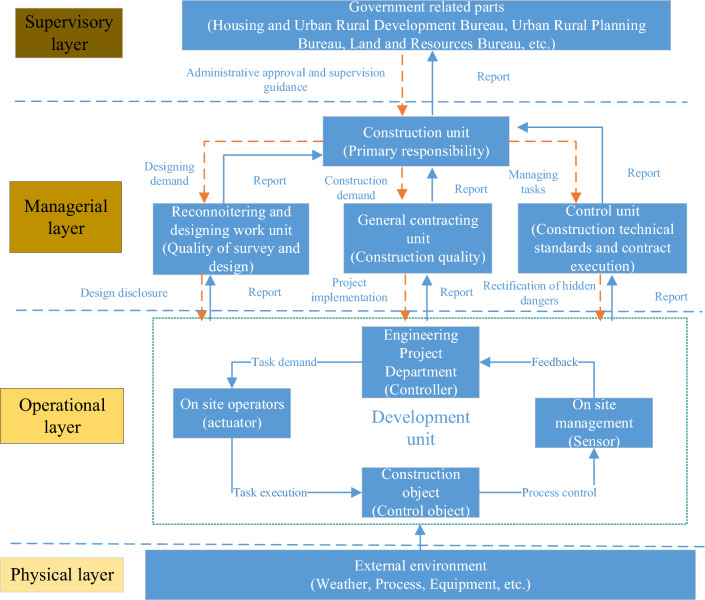
Control structure of a building collapse accident.

Construction engineering safety control is the basis for analyzing accident causes using the STAMP model. Based on the actual operation of China's construction projects, the safety control structure is divided into a supervisory layer, a managerial layer, an operational layer, and a physical layer (Fig. 4).

1. The supervisory level includes government supervision departments and supervision units. A government supervision department controls the safety management of the construction industry as a whole through legislation and by issuing norms and standards. A supervision unit accepts the entrustment demand of a construction unit and carries out safety production supervision and hidden accident danger rectification at a construction site.
2. The managerial level includes all units participating in a construction project and adopts an organizational form that establishes a project department to make all participating parties coordinate with and restrict each other and to control the safety and progress of the project. A construction unit is the investor and manager of a construction project, which realizes the constraints by describing the needs to other participating units and receiving feedback from them. The survey and design units accept the entrustment needs of a construction unit, and they provide it with true and accurate survey and design documents. The construction unit controls the whole process of project progress, quality management and safety management at a construction site.
3. The operational layer includes the operators and team leaders at a construction site. The operators accept the instructions and constraints of a construction unit and construct based on the construction organization plan and safety rules and regulations^32^. At the same time, the construction unit restricts the operators through on-site inspection, training and education.
4. The physical layer includes mechanical equipment, materials and the construction environment^33^. Front-line employees may encounter interference from various factors in the physical layer during actual construction operations.

By analyzing the investigation reports on 355 building collapse accidents and combining them with the control structure chart of building collapse accidents, starting from the physical layer, operational layer, managerial layer and supervisory layer, 22 main influencing factors as well as the level and main performance of each factor are finally obtained (Table 2).

**Table 2:** Analysis of the factors causing building collapse accidents based on the STAMP model.

System level	Control structure	Causal factor	Typical manifestation
Physical layer	Mechanical equipment	Unqualified building materials S_1_	Insufficient strength of materials such as concrete, cement, and steel
		Construction equipment failure S_2_	Faults in cranes, tower cranes, concrete mixer trucks, excavators, protective equipment, etc
	Environment	Soil resources S_3_	Insufficient land bearing capacity, uneven soil settlement, changes in the groundwater level, etc
		Surrounding buildings S_4_	Poor housing, lighting, ventilation, and road conditions around the construction site
		Extreme weather S_5_	Rainstorms, typhoons, high temperatures, etc
Operational layer	Front-line staff	No job qualification S_6_	Work without holding relevant professional qualification certificates, etc
		Illegal work S_7_	Failure to wear proper safety equipment, violation of safety operation regulations, etc
		Not skilled in operation S_8_	Insufficient safety awareness, unfamiliarity with operating procedures, etc
		Poor health S_9_	Suffering from fainting, blurred consciousness, dizziness, etc
	Squad leader	Illegal command S_10_	Lack of decision-making ability, lucky psychology, etc
		Error of judgment S_11_	Lack of on-site management experience, influenced by decisions made by others, etc
Managerial layer	Project manager	Insufficient safety awareness S_12_	Incomplete and inadequate identification of hidden dangers
		Insufficient safety training S_13_	Inadequate safety training time and quality
		Unreasonable construction plan S_14_	Failure to prepare a construction plan before construction, resulting in chaotic construction procedures
	Business executives	Safety policy not implemented S_15_	Inadequate implementation of safety policies or documents issued by the government
		Insufficient safety investment S_16_	Safety funds have been misappropriated, with insufficient investment effectiveness, etc
		Loose safety management S_17_	Lack of effective safety management activities, systems, and methods
Supervisory layer	Supervision department	Untimely rectification feedback S_18_	Failure to review within the valid time frame
		Insufficient inspection work S_19_	Inability to effectively identify hidden danger points
	Housing and urban‒rural development sector	Benefit exchange behavior S_20_	Exchange of interests between the regulator and the regulated
		Dereliction of duty in supervision S_21_	Failure to fulfill regulatory responsibilities for the construction market, construction activities, and engineering quality
		Incomplete policies and systems S_22_	Loopholes in regulatory systems, lack of enforcement, etc

*S*_*ij*_* is the symbol for the causal factors of building collapse accidents.*

### Using the triangular fuzzy DEMATEL model to analyze the interrelationships between the causal factors of collapse accidents

We invited seven experts to score the influence strength among the 22 factors and constructed a corresponding evaluation score matrix. Based on the corresponding relationship in Table 1, a triangular fuzzy evaluation variable matrix was obtained, and the results were calculated based on solution steps (2)-(5) of the triangular fuzzy DEMATEL method.

The initial direct matrix Z (Appendix 1) of building collapse accidents was calculated using formulas (1)-(8).

The comprehensive action matrix *F* of building collapse accidents was calculated using formulas (9)-(11) (Appendix 2).

The influence degree, affected degree, center degree and cause degree of the factors causing a building collapse accident were obtained using formulas (12)-(15) (Table 3). The higher the centrality value is, the greater the impact of representative factors on building collapse accidents, and vice versa. There are positive and negative cause degrees. A positive cause degree indicates a stronger influence on other causal factors, whereas a negative cause degree indicates that the causal factor is strongly affected by other factors.

**Table 3:** Influence degree, affected degree, center degree and cause degree of building collapse accidents.

Index	Affected degree	Influence degree	Center degree	Centrality ranking	Cause degree	Indicator properties
S_1_	0.88	0.64	1.52	18	-0.23	Result
S_2_	1.08	0.57	1.66	15	-0.51	Result
S_3_	1.12	0.55	1.67	12	-0.56	Result
S_4_	0.88	0.74	1.61	16	-0.14	Result
S_5_	1.18	0.63	1.81	6	-0.55	Result
S_6_	0.83	0.47	1.29	21	-0.36	Result
S_7_	1.12	0.65	1.77	9	-0.47	Result
S_8_	0.64	0.70	1.34	20	0.06	Reason
S_9_	1.20	0.62	1.81	7	-0.58	Result
S_10_	1.35	0.78	2.13	2	-0.57	Reason
S_11_	1.54	0.48	2.02	5	-1.05	Reason
S_12_	0.65	1.01	1.66	14	0.36	Reason
S_13_	1.22	0.81	2.02	4	-0.41	Result
S_14_	0.52	0.84	1.36	19	0.31	Reason
S_15_	0.81	0.87	1.69	10	0.06	Reason
S_16_	0.68	0.90	1.58	17	0.22	Reason
S_17_	0.57	1.77	2.34	1	1.20	Reason
S_18_	0.61	1.41	2.02	3	0.80	Reason
S_19_	0.55	1.23	1.78	8	0.68	Reason
S_20_	0.46	1.21	1.67	13	0.74	Reason
S_21_	0.48	1.20	1.68	11	0.72	Reason
S_22_	0.46	0.75	1.21	22	0.28	Reason

**Figure 5:**
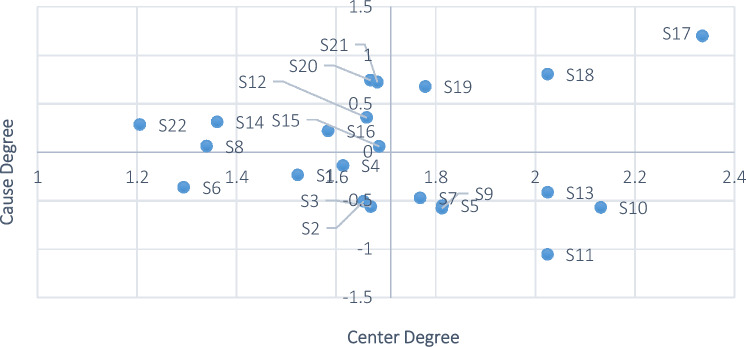
Quadrant distribution of the center degree and cause degree of building collapse accidents.

Based on the data in Table 3, we used the quadrant determination method to draw a causal relationship diagram with centrality and causality as the horizontal and vertical axes, respectively, and we marked the position of each element. The intersection of the horizontal and vertical coordinates is *O* (1.71, 0) (1.71 is the average of the sum of the center degrees). The 22 factors identified were plotted in four quadrants based on the quadrant determination method described above (Fig. 5).

### ***Hierarchical analysis ***of*** the causal factors of building collapse accidents based on ISM***

Based on the analysis of the causality and centrality of building collapse accidents using the DEMATEL method, ISM was further used to explore the hierarchical structure and overall influence relationship of the causal factors of building collapse accidents. Using formulas (16)-(17), we calculated the reachability matrix *K* of the causal factors of building collapse accidents (Appendix 3).

Based on the reachability matrix *K* of the causal factors of building collapse accidents, we used formulas (17)-(19) to calculate reachability set *R(S*_*i*_*),* antecedent set *A(Si)* and common set *C(Si)*. The reachability set is the set of the 1 row where the causal factor is located, the antecedent set is the set of the 1 column where the causal factor is located, and the common set is the intersection of the reachability set and the antecedent set (Table 4).

**Table 4:** Reachability set, antecedent set and common set of building collapse accidents.

	R(S_i_)	A(S_i_)	C(S_i_)
S_1_	1,11	1,12,13,15–20,22	1
S_2_	2,11	2,10,12,13,16–21	2
S_3_	3,11	3,10,12,13,16–21	3
S_4_	4,5,11	4,10,12,13,16–21	4
S_5_	5,10	4,5,10,12,13,16–20	5,10
S_6_	6	6,17,18,20,22	6
S_7_	7	7,8,10,12–14,16–21	7
S_8_	7,8	8,10,12–14,16–20	8
S_9_	9	4,9,10,12,13,16–22	9
S_10_	2,3,4,5,7,9,10,11	10,12,13,16–21	10
S_11_	11	1–5,10–13,15–22	11
S_12_	1–5,7–13,16	12,13,16–20	12,13,16
S_13_	1–5,7–13,16	1,12,13,16–22	12,13,16
S_14_	7,8,14	14,17	14
S_15_	1,9,11,15	15–22	15
S_16_	1–5,7–13,16	13,14,16–20	13,16
S_17_	1–19	17	17
S_18_	1–13,16,18,19	17,18,20	18
S_19_	1–5,7–13,16,19	17–20	19
S_20_	1–13,15,16,18–22	20	20
S_21_	2–5,7,9–11,21	20, 21	21
S_22_	1,6,9,11,15,22	20, 22	22

**Figure 6:**
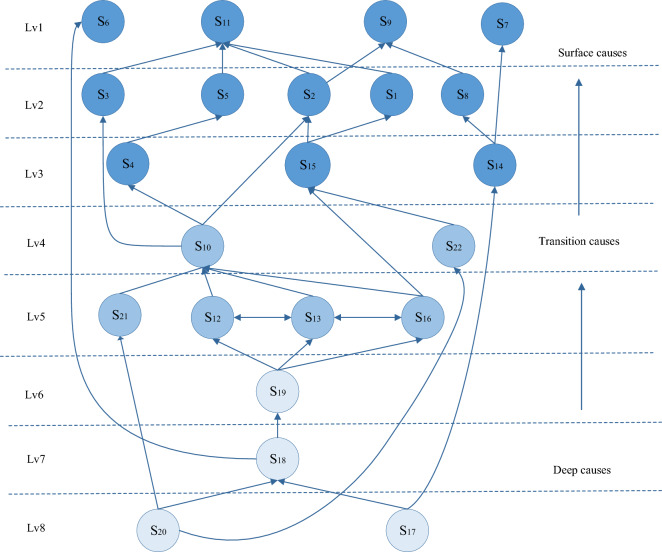
ISM hierarchy of the causal factors of building collapse accidents.

Using the *R(Si)* = *C(Si)* criterion, the first layers of the causal factors of building collapse accidents are S_6,_ S_7_, S_9_, and S_11_. The first-level factors are removed from the reachability set, antecedent set and common set. Then, based on this criterion, the second-level factors of the causal factors of building collapse accidents are S_1_, S_2_, S_3_, S_5_, and S_8_. By iteratively repeating this method, a multilevel ISM model of the causal factors of building collapse accidents is finally obtained (Fig. 6).

### Result analysis

By using the DEMATEL method, the influence degree, affected degree, cause degree and center degree of the factors causing building collapse accidents are determined, and the key factors are identified. In addition, a multilayer hierarchical structure model of building collapse accidents is constructed using the ISM method to reveal the direct, indirect and deep-rooted causes of building collapse accidents. We obtain the following results:

(1) Analysis of the influence degree and affected degree.

As shown in Table 3, the most influential factor is loose safety management (S_17_) at the managerial level, followed by untimely rectification feedback (S_18_), ineffective inspection work (S_19_), benefit exchange behavior (S_20_), and dereliction of duty in supervision (S_21_) at the supervisory level. In addition, the factors insufficient safety awareness (S_12_) and insufficient safety investment (S_16_) at the managerial level are highly influential. The most affected factor is error of judgment (S_11_), followed by illegal command (S_10_) and insufficient safety training (S_13_). Poor health (S_9_), illegal work (S_7_) and extreme weather (S_5_) also have a high influence degree. These results show that in building construction, the control structures that are easily affected are mainly at the operational layer and the physical layer. These factors are mainly affected by corporate management and government regulation. By strengthening the supervision of various government departments and improving the level of enterprise safety management and investment in safety production, managers can increase the safety management of construction sites, discover potential safety hazards in time, and employ front-line operators in accordance with safety regulations to ensure the standardization and stability of the support system.

(2) Analysis of the center degree and cause degree.

The top three factors of the center degree are loose safety management (S_17_), illegal command (S_10_) and untimely rectification feedback (S_18_). These three factors are the most important in the causal system of building collapse accidents and play the most obvious role in the occurrence of such accidents. Therefore, to prevent the occurrence of building collapse accidents, we need to focus on controlling the occurrence of these three factors.

Figure 5 shows that the center degree and cause degree of factors S_17_, S_18_ and S_19_ in the first quadrant are high, indicating that they are the key factors affecting building collapse accidents and need to be treated and improved first. Factors S_5_, S_7_, S_9_-S_11_ and S_13_ in the second quadrant have a high center degree but a low cause degree. They are supportive factors and play an auxiliary role in the model. There are five factors in the third quadrant, S_1_-S_4_ and S_6_. Both their cause degree and centrality are low, they are independent factors, and they are vulnerable to other factors. Factors S_8_, S_12_, S_14_-S_16_ and S_20_-S_22_ are in the fourth quadrant. Although the center degree of these factors is slightly lower, the cause degree is higher, which means that they are core problem elements. Although they are not key factors affecting building collapse accidents, they have a strong impact on other factors. Therefore, these factors still need to be considered.

(3) Analysis of the ISM hierarchy.

Based on Fig. 6, the causal factors of building collapse accidents include surface causes, intermediate causes and deep-rooted causes, which are divided into eight levels. Among them, S_6_, S_7_, S_9_ and S_11_ are the surface causes of building collapse accidents. The factors of other layers are directly or indirectly related to these four factors, which shows that these problems can be solved by improving the factors of other layers. For example, we can organize safety education and training through enterprises to ensure that staff are familiar with safe operation technologies and procedures and have sufficient safety knowledge and safety awareness to prevent accidents caused by illegal operations. We can also prevent accidents by strengthening the supervision of safety management departments to ensure that staff operate or command based on the specifications.

There are 14 factors in the intermediate layers (layers 2–5) of the system, namely, S_1_-S_5_, S_8_, S_10_, S_12_-S_16_, S_21_ and S_22_. Among them, the lowest contributing factors are insufficient safety awareness (S_12_), inadequate safety training (S_13_), insufficient safety investment (S_16_) and dereliction of duty in supervision (S_21_). In addition, the interaction of S_12_, S_13_ and S_16_ will strengthen the influence of the intermediate layers on the surface layer. Although the causal factors of the intermediate layers are not direct influencing factors or deep-rooted influencing factors, they also indirectly affect the occurrence of building collapse accidents, which cannot be ignored.

The causal factors (layers 6–8) located in the deep-rooted layers of the system include four factors: S_17_, S_18_, S_19_ and S_20_. The deep-rooted influencing factors affect the surface influencing factors by affecting the intermediate factors. The lowest of the deep-rooted influencing factors involve two aspects: loose safety management (S_17_) at the managerial level and benefit exchange behavior (S_20_) at the supervisory level. These two factors jointly impact the timeliness of rectification feedback from government departments (S_18_) to affect the intermediate influencing factors. In addition, S_17_ will also directly affect the unreasonable construction plan at the intermediate layers (S_14_), and S_20_ will directly affect dereliction of duty in supervision (S_21_) to exert an impact on the surface causal factors.

## Discussion

With the continuous innovation of technology and the continuous development of the economy, in terms of both the building volume and technological complexity, the risks in the construction process have increased significantly compared to any previous period, resulting in higher levels of accidents. The increase in the complexity of accident causes has led to deviations in the applicability of traditional accident cause theories. Traditional accident cause theories divide the causes of building collapse accidents into human, equipment, environmental and managerial factors^34,35^. Although this method can comprehensively identify the causal factors of accidents, it is too loose for controlling accidents. The STAMP method based on system theory is based on the control structure, which allows a site manager to determine the cause of a collapse accident more logically.

For each system safety constraint, determining whether the high-level system has assigned responsibility for executing the constraint to the lower components in the security control structure or whether the high-level system has sufficient control to ensure that the assigned security constraint is implemented in the lower components is the key to analyzing the cause of the accident^36^. Through the dynamic perspective of the STAMP model, it was found that the lack of communication and coordination among various levels of collapse accidents as well as feedback loops (safety inspection reports, maintenance feedback, etc.) are key factors leading to incorrect decision-making or inappropriate control^37^. The upper level is not aware of the true situation of the lower level. As a result, it is impossible to determine whether the system's safety margin is sufficient, and as a result, local adjustments made by superiors are no longer effective.

In the study of building collapse accidents, most of the research results point to worker violations as the direct cause of such accidents^38,39^. Clearly, this problem is also a key point that we obtain from the DEMATEL-ISM model. However, the more organizational reasons underlying this problem are the key to preventing collapse accidents. Loose enterprise safety management will affect managers' failure to pay attention to the management and safety training of front-line personnel. In addition, enterprises will not pay attention to investing in safety. Another problem that is easy to ignore is a problem in the regulatory process, especially the exchange of interests between government departments and enterprises^40^.

This interest exchange includes two forms. First, enterprises influence the government's regulatory decisions through their own influence. However, based on the actual situation in China, local governments still play a leading role in the exchange relationship between local governments and enterprises, which can lead to laziness in enterprise safety work and dependence on government regulatory authorities. However, with the deepening of reform and the acceleration of market-oriented processes, the influence of enterprises is also increasing. Second, not all levels of government departments have established a complete management mechanism for the safety supervision of construction projects^41^. The combination of multiple departments, such as safety production supervision and management, quality and technical supervision, environmental protection, land and resources, urban and rural planning, and fire protection departments, makes it difficult to form an effective and diverse collection, resulting in management loopholes and delayed supervision of hidden danger rectification.

To prevent and control construction collapse accidents, targeted preventive and control measures should be proposed by considering the attributes of various levels of factors. Resolving problems in the operational layer relies on other factors. For instance, in addressing the issue of front-line workers engaging in unauthorized operations, the managerial layer should organize safety education and training programs to enhance these workers’ operational competence and safety awareness. Regarding problems in the physical layer, the supervisory layer should intensify technological innovation and research and development efforts to enhance construction process standards. Each unit within the managerial level should also implement the system of safety production responsibility to fundamentally ensure the level of project safety management. The supervisory layer should enforce organizational construction standards, strengthen law enforcement efforts, and intensify penalties for violations to address the deep-rooted causes of accidents.

## Conclusions

Based on investigation reports on 355 typical building collapse accidents, this paper uses the STAMP method to extract the causes of such accidents from the four control levels of the physical layer, field operational layer, enterprise managerial layer and government supervisory layer. Through a literature analysis and questionnaire survey of relevant experts, 22 kinds of factors that cause building collapse accidents are finally determined. Using an improved DEMATEL-ISM method, the logical relationship between the factors influencing building collapse accidents is analyzed in depth. Most of the factors in the physical layer and operational layer are result elements that are vulnerable to managerial and supervisory factors. The deep-rooted reasons for collapse accidents are mainly loose safety management at the managerial layer and the exchange of interests at the supervisory layer. Through the influence on the causal factors of the intermediate layers, these factors impact workers and managers at the operational layer and equipment and the environment at the physical layer.

Combining the STAMP method with the triangular fuzzy DEMATEL-ISM method to analyze the causal factors of building collapse accidents can address not only the defect of a lack of focus in STAMP method analysis but also the limitation of relationship classification of any two factors in the ISM method to further analyze the logical relationship and influence intensity between accident influencing factors. However, this paper still has the following limitations. First, the statistical sample size for building collapse accidents is relatively small. Without comprehensive government statistics on building collapse accidents, there may be certain omissions when analyzing their causes. Second, while the modularized STAMP method can analyze a broad range of building collapse accidents, it may not sufficiently cover the intricacies of specific types of collapse incidents. Finally, the analysis of the causes of building collapse accidents does not account for the influence of the occurrence probability of different collapse accident types and their influencing factors.

Therefore, in future research, we will endeavor to collaborate with the government in establishing a standardized accident statistics database^42^. Additionally, we will integrate accident causality models with machine learning, statistical principles, and other methodologies to achieve more precise control over accidents^43^. Furthermore, this paper uses only part of the text data on an accident, and there is still a large amount of halo data information in the form of pictures and videos that is not used. We will focus on protocol processing, database establishment and data mining of this information.

### Supplementary Information

Supplementary Information.

## Data Availability

All data generated or analyzed during this study are included in this published article.
