# Supplementary material for: A hybrid STAMP-fuzzy DEMATEL-ISM approach for analyzing the factors influencing building collapse accidents in China
Source: Sci Rep. 2023 Nov 13;13:19745. doi: 10.1038/s41598-023-46778-6 (PMC10643550; doi:10.1038/s41598-023-46778-6)
Supplement: Supplementary file 1 — Supplementary Information. [file 41598_2023_46778_MOESM1_ESM.docx]

**Appendix**

**Appendix 1.** Initial direct matrix *Z* of building collapse accidents

| Index | S_1_ | S_2_ | S_3_ | S_4_ | S_5_ | S_6_ | S_7_ | S_8_ | S_9_ | S_10_ | S_11_ | S_12_ | S_13_ | S_14_ | S_15_ | S_16_ | S_17_ | S_18_ | S_19_ | S_20_ | S_21_ | S_22_ |
| --- | --- | --- | --- | --- | --- | --- | --- | --- | --- | --- | --- | --- | --- | --- | --- | --- | --- | --- | --- | --- | --- | --- |
| S_1_ | 0 | 0.04 | 0.04 | 0.33 | 0.33 | 0.04 | 0.04 | 0.04 | 0.17 | 0.17 | 0.57 | 0.13 | 0.04 | 0.07 | 0.04 | 0.17 | 0.04 | 0.04 | 0.04 | 0.17 | 0.04 | 0.17 |
| S_2_ | 0.04 | 0 | 0.04 | 0.23 | 0.43 | 0.27 | 0.04 | 0.04 | 0.23 | 0.07 | 0.43 | 0.04 | 0.1 | 0.04 | 0.04 | 0.04 | 0.04 | 0.04 | 0.04 | 0.17 | 0.04 | 0.04 |
| S_3_ | 0.07 | 0.1 | 0 | 0.04 | 0.37 | 0.04 | 0.13 | 0.1 | 0.04 | 0.04 | 0.43 | 0.1 | 0.2 | 0.17 | 0.04 | 0.04 | 0.04 | 0.04 | 0.04 | 0.17 | 0.07 | 0.04 |
| S_4_ | 0.04 | 0.23 | 0.4 | 0 | 0.47 | 0.04 | 0.04 | 0.17 | 0.7 | 0.04 | 0.43 | 0.04 | 0.13 | 0.04 | 0.04 | 0.04 | 0.04 | 0.04 | 0.04 | 0.17 | 0.04 | 0.04 |
| S_5_ | 0.04 | 0.1 | 0.07 | 0.07 | 0 | 0.04 | 0.1 | 0.04 | 0.04 | 0.04 | 0.43 | 0.04 | 0.3 | 0.04 | 0.04 | 0.17 | 0.17 | 0.17 | 0.17 | 0.17 | 0.17 | 0.04 |
| S_6_ | 0.04 | 0.07 | 0.04 | 0.33 | 0.04 | 0 | 0.47 | 0.04 | 0.04 | 0.17 | 0.04 | 0.04 | 0.23 | 0.04 | 0.04 | 0.04 | 0.04 | 0.04 | 0.04 | 0.04 | 0.04 | 0.04 |
| S_7_ | 0.07 | 0.07 | 0.1 | 0.33 | 0.2 | 0.47 | 0 | 0.04 | 0.33 | 0.17 | 0.07 | 0.07 | 0.33 | 0.13 | 0.1 | 0.04 | 0.04 | 0.04 | 0.04 | 0.04 | 0.04 | 0.04 |
| S_8_ | 0.04 | 0.33 | 0.33 | 0.2 | 0.2 | 0.13 | 0.47 | 0 | 0.37 | 0.27 | 0.1 | 0.07 | 0.07 | 0.1 | 0.07 | 0.04 | 0.04 | 0.04 | 0.04 | 0.04 | 0.04 | 0.04 |
| S_9_ | 0.07 | 0.04 | 0.27 | 0.13 | 0.4 | 0.17 | 0.2 | 0.04 | 0 | 0.23 | 0.1 | 0.04 | 0.23 | 0.13 | 0.27 | 0.04 | 0.04 | 0.04 | 0.04 | 0.04 | 0.04 | 0.04 |
| S_10_ | 0.04 | 0.47 | 0.47 | 0.47 | 0.23 | 0.1 | 0.47 | 0.07 | 0.3 | 0 | 0.17 | 0.04 | 0.07 | 0.04 | 0.2 | 0.04 | 0.04 | 0.04 | 0.04 | 0.04 | 0.04 | 0.04 |
| S_11_ | 0.07 | 0.33 | 0.04 | 0.04 | 0.07 | 0.1 | 0.1 | 0.04 | 0.33 | 0.4 | 0 | 0.04 | 0.07 | 0.04 | 0.07 | 0.04 | 0.04 | 0.04 | 0.04 | 0.04 | 0.04 | 0.04 |
| S_12_ | 0.04 | 0.33 | 0.33 | 0.13 | 0.33 | 0.07 | 0.07 | 0.04 | 0.1 | 0.2 | 0.04 | 0 | 0.43 | 0.23 | 0.27 | 0.6 | 0.1 | 0.17 | 0.33 | 0.04 | 0.13 | 0.04 |
| S_13_ | 0.07 | 0.1 | 0.04 | 0.07 | 0.04 | 0.07 | 0.07 | 0.73 | 0.1 | 0.2 | 0.04 | 0.6 | 0 | 0.04 | 0.33 | 0.1 | 0.1 | 0.13 | 0.17 | 0.07 | 0.04 | 0.04 |
| S_14_ | 0.33 | 0.33 | 0.33 | 0.33 | 0.04 | 0.07 | 0.23 | 0.73 | 0.17 | 0.2 | 0.1 | 0.13 | 0.13 | 0 | 0.17 | 0.04 | 0.04 | 0.04 | 0.04 | 0.04 | 0.04 | 0.04 |
| S_15_ | 0.6 | 0.04 | 0.04 | 0.04 | 0.04 | 0.13 | 0.04 | 0.04 | 0.73 | 0.3 | 0.3 | 0.1 | 0.07 | 0.04 | 0 | 0.4 | 0.4 | 0.04 | 0.04 | 0.04 | 0.04 | 0.04 |
| S_16_ | 0.6 | 0.04 | 0.04 | 0.04 | 0.04 | 0.04 | 0.04 | 0.04 | 0.17 | 0.57 | 0.6 | 0.13 | 0.7 | 0.07 | 0.27 | 0 | 0.17 | 0.04 | 0.04 | 0.04 | 0.04 | 0.04 |
| S_17_ | 0.07 | 0.33 | 0.73 | 0.37 | 0.33 | 0.04 | 0.73 | 0.1 | 0.13 | 0.74 | 0.74 | 0.6 | 0.74 | 0.4 | 0.4 | 0.4 | 0 | 0.4 | 0.04 | 0.04 | 0.04 | 0.04 |
| S_18_ | 0.04 | 0.33 | 0.6 | 0.04 | 0.04 | 0.33 | 0.6 | 0.04 | 0.23 | 0.6 | 0.67 | 0.13 | 0.6 | 0.07 | 0.17 | 0.1 | 0.1 | 0 | 0.6 | 0.33 | 0.1 | 0.17 |
| S_19_ | 0.33 | 0.33 | 0.33 | 0.04 | 0.33 | 0.33 | 0.04 | 0.04 | 0.2 | 0.74 | 0.67 | 0.07 | 0.4 | 0.04 | 0.17 | 0.04 | 0.4 | 0.4 | 0 | 0.04 | 0.04 | 0.04 |
| S_20_ | 0.6 | 0.04 | 0.04 | 0.04 | 0.04 | 0.13 | 0.04 | 0.04 | 0.04 | 0.04 | 0.04 | 0.07 | 0.07 | 0.07 | 0.07 | 0.4 | 0.4 | 0.4 | 0.4 | 0 | 0.73 | 0.73 |
| S_21_ | 0.33 | 0.73 | 0.33 | 0.04 | 0.73 | 0.33 | 0.73 | 0.04 | 0.1 | 0.43 | 0.3 | 0.07 | 0.04 | 0.04 | 0.1 | 0.04 | 0.04 | 0.4 | 0.04 | 0.04 | 0 | 0.27 |
| S_22_ | 0.33 | 0.04 | 0.07 | 0.1 | 0.04 | 0.47 | 0.04 | 0.04 | 0.2 | 0.1 | 0.1 | 0.04 | 0.13 | 0.33 | 0.47 | 0.07 | 0.07 | 0.07 | 0.04 | 0.04 | 0.27 | 0 |

**Appendix 2.** Comprehensive impact matrix *F* of building collapse accidents

|  | S_1_ | S_2_ | S_3_ | S_4_ | S_5_ | S_6_ | S_7_ | S_8_ | S_9_ | S_10_ | S_11_ | S_12_ | S_13_ | S_14_ | S_15_ | S_16_ | S_17_ | S_18_ | S_19_ | S_20_ | S_21_ | S_22_ |
| --- | --- | --- | --- | --- | --- | --- | --- | --- | --- | --- | --- | --- | --- | --- | --- | --- | --- | --- | --- | --- | --- | --- |
| S_1_ | 0.01 | 0.02 | 0.02 | 0.06 | 0.06 | 0.02 | 0.02 | 0.01 | 0.04 | 0.04 | 0.1 | 0.03 | 0.02 | 0.02 | 0.02 | 0.03 | 0.01 | 0.01 | 0.01 | 0.03 | 0.01 | 0.03 |
| S_2_ | 0.02 | 0.01 | 0.02 | 0.04 | 0.07 | 0.05 | 0.02 | 0.01 | 0.05 | 0.03 | 0.08 | 0.01 | 0.03 | 0.01 | 0.02 | 0.01 | 0.01 | 0.01 | 0.01 | 0.03 | 0.01 | 0.01 |
| S_3_ | 0.02 | 0.03 | 0.01 | 0.02 | 0.06 | 0.02 | 0.03 | 0.02 | 0.02 | 0.02 | 0.08 | 0.02 | 0.04 | 0.03 | 0.02 | 0.01 | 0.01 | 0.01 | 0.01 | 0.03 | 0.02 | 0.01 |
| S_4_ | 0.02 | 0.05 | 0.07 | 0.01 | 0.08 | 0.02 | 0.02 | 0.03 | 0.11 | 0.03 | 0.08 | 0.02 | 0.04 | 0.01 | 0.02 | 0.02 | 0.02 | 0.02 | 0.02 | 0.03 | 0.01 | 0.01 |
| S_5_ | 0.02 | 0.03 | 0.03 | 0.02 | 0.02 | 0.02 | 0.03 | 0.02 | 0.02 | 0.03 | 0.08 | 0.02 | 0.06 | 0.01 | 0.02 | 0.03 | 0.03 | 0.03 | 0.03 | 0.03 | 0.03 | 0.01 |
| S_6_ | 0.01 | 0.02 | 0.02 | 0.06 | 0.02 | 0.01 | 0.07 | 0.01 | 0.02 | 0.04 | 0.02 | 0.01 | 0.04 | 0.01 | 0.01 | 0.01 | 0.01 | 0.01 | 0.01 | 0.01 | 0.01 | 0.01 |
| S_7_ | 0.02 | 0.02 | 0.03 | 0.06 | 0.05 | 0.07 | 0.02 | 0.02 | 0.06 | 0.04 | 0.03 | 0.02 | 0.06 | 0.02 | 0.03 | 0.01 | 0.01 | 0.01 | 0.01 | 0.01 | 0.01 | 0.01 |
| S_8_ | 0.02 | 0.06 | 0.06 | 0.04 | 0.05 | 0.03 | 0.08 | 0.01 | 0.07 | 0.05 | 0.04 | 0.02 | 0.03 | 0.02 | 0.02 | 0.01 | 0.01 | 0.01 | 0.01 | 0.01 | 0.01 | 0.01 |
| S_9_ | 0.02 | 0.02 | 0.05 | 0.03 | 0.07 | 0.03 | 0.04 | 0.02 | 0.02 | 0.05 | 0.04 | 0.02 | 0.05 | 0.02 | 0.05 | 0.02 | 0.01 | 0.01 | 0.01 | 0.01 | 0.01 | 0.01 |
| S_10_ | 0.02 | 0.08 | 0.08 | 0.08 | 0.06 | 0.03 | 0.08 | 0.02 | 0.07 | 0.02 | 0.05 | 0.02 | 0.03 | 0.02 | 0.04 | 0.02 | 0.01 | 0.01 | 0.01 | 0.02 | 0.01 | 0.01 |
| S_11_ | 0.02 | 0.06 | 0.02 | 0.02 | 0.03 | 0.02 | 0.03 | 0.01 | 0.06 | 0.07 | 0.02 | 0.01 | 0.02 | 0.01 | 0.02 | 0.01 | 0.01 | 0.01 | 0.01 | 0.01 | 0.01 | 0.01 |
| S_12_ | 0.03 | 0.07 | 0.07 | 0.04 | 0.07 | 0.03 | 0.03 | 0.03 | 0.04 | 0.06 | 0.05 | 0.02 | 0.09 | 0.04 | 0.06 | 0.1 | 0.03 | 0.04 | 0.06 | 0.02 | 0.03 | 0.01 |
| S_13_ | 0.03 | 0.04 | 0.03 | 0.03 | 0.03 | 0.03 | 0.03 | 0.11 | 0.04 | 0.05 | 0.03 | 0.09 | 0.03 | 0.02 | 0.06 | 0.03 | 0.03 | 0.03 | 0.03 | 0.02 | 0.01 | 0.01 |
| S_14_ | 0.06 | 0.07 | 0.07 | 0.06 | 0.03 | 0.03 | 0.05 | 0.11 | 0.05 | 0.05 | 0.04 | 0.03 | 0.04 | 0.01 | 0.04 | 0.02 | 0.01 | 0.01 | 0.01 | 0.02 | 0.01 | 0.01 |
| S_15_ | 0.1 | 0.03 | 0.03 | 0.03 | 0.03 | 0.03 | 0.03 | 0.02 | 0.12 | 0.07 | 0.08 | 0.03 | 0.04 | 0.02 | 0.02 | 0.07 | 0.06 | 0.02 | 0.01 | 0.01 | 0.01 | 0.01 |
| S_16_ | 0.1 | 0.03 | 0.03 | 0.03 | 0.03 | 0.02 | 0.03 | 0.02 | 0.05 | 0.1 | 0.11 | 0.04 | 0.11 | 0.02 | 0.05 | 0.02 | 0.03 | 0.02 | 0.02 | 0.02 | 0.01 | 0.01 |
| S_17_ | 0.04 | 0.09 | 0.14 | 0.09 | 0.09 | 0.04 | 0.14 | 0.05 | 0.07 | 0.15 | 0.16 | 0.11 | 0.15 | 0.07 | 0.09 | 0.08 | 0.02 | 0.07 | 0.03 | 0.03 | 0.02 | 0.02 |
| S_18_ | 0.03 | 0.08 | 0.11 | 0.04 | 0.05 | 0.08 | 0.11 | 0.03 | 0.07 | 0.13 | 0.14 | 0.04 | 0.12 | 0.03 | 0.05 | 0.03 | 0.03 | 0.02 | 0.1 | 0.06 | 0.03 | 0.04 |
| S_19_ | 0.06 | 0.08 | 0.08 | 0.04 | 0.08 | 0.07 | 0.04 | 0.02 | 0.06 | 0.14 | 0.14 | 0.03 | 0.09 | 0.02 | 0.05 | 0.02 | 0.07 | 0.07 | 0.02 | 0.02 | 0.02 | 0.02 |
| S_20_ | 0.11 | 0.04 | 0.04 | 0.03 | 0.04 | 0.05 | 0.04 | 0.02 | 0.04 | 0.05 | 0.06 | 0.03 | 0.05 | 0.03 | 0.04 | 0.07 | 0.07 | 0.08 | 0.07 | 0.01 | 0.11 | 0.11 |
| S_21_ | 0.06 | 0.13 | 0.07 | 0.03 | 0.13 | 0.07 | 0.13 | 0.02 | 0.05 | 0.09 | 0.09 | 0.02 | 0.04 | 0.02 | 0.03 | 0.02 | 0.02 | 0.07 | 0.02 | 0.02 | 0.01 | 0.05 |
| S_22_ | 0.06 | 0.02 | 0.03 | 0.03 | 0.03 | 0.08 | 0.03 | 0.02 | 0.05 | 0.04 | 0.04 | 0.02 | 0.04 | 0.05 | 0.08 | 0.02 | 0.02 | 0.02 | 0.01 | 0.01 | 0.04 | 0.01 |

**Appendix 3.** Reachability matrix *K* of the causal factors of building collapse accidents

| Index | S_1_ | S_2_ | S_3_ | S_4_ | S_5_ | S_6_ | S_7_ | S_8_ | S_9_ | S_10_ | S_11_ | S_12_ | S_13_ | S_14_ | S_15_ | S_16_ | S_17_ | S_18_ | S_19_ | S_20_ | S_21_ | S_22_ |
| --- | --- | --- | --- | --- | --- | --- | --- | --- | --- | --- | --- | --- | --- | --- | --- | --- | --- | --- | --- | --- | --- | --- |
| S_1_ | 1 | 0 | 0 | 0 | 0 | 0 | 0 | 0 | 0 | 0 | 1 | 0 | 0 | 0 | 0 | 0 | 0 | 0 | 0 | 0 | 0 | 0 |
| S_2_ | 0 | 1 | 0 | 0 | 0 | 0 | 0 | 0 | 0 | 0 | 1 | 0 | 0 | 0 | 0 | 0 | 0 | 0 | 0 | 0 | 0 | 0 |
| S_3_ | 0 | 0 | 1 | 0 | 0 | 0 | 0 | 0 | 0 | 0 | 1 | 0 | 0 | 0 | 0 | 0 | 0 | 0 | 0 | 0 | 0 | 0 |
| S_4_ | 0 | 0 | 0 | 1 | 1 | 0 | 0 | 0 | 1 | 0 | 1 | 0 | 0 | 0 | 0 | 0 | 0 | 0 | 0 | 0 | 0 | 0 |
| S_5_ | 0 | 0 | 0 | 0 | 1 | 0 | 0 | 0 | 0 | 0 | 1 | 0 | 0 | 0 | 0 | 0 | 0 | 0 | 0 | 0 | 0 | 0 |
| S_6_ | 0 | 0 | 0 | 0 | 0 | 1 | 0 | 0 | 0 | 0 | 0 | 0 | 0 | 0 | 0 | 0 | 0 | 0 | 0 | 0 | 0 | 0 |
| S_7_ | 0 | 0 | 0 | 0 | 0 | 0 | 1 | 0 | 0 | 0 | 0 | 0 | 0 | 0 | 0 | 0 | 0 | 0 | 0 | 0 | 0 | 0 |
| S_8_ | 0 | 0 | 0 | 0 | 0 | 0 | 1 | 1 | 0 | 0 | 0 | 0 | 0 | 0 | 0 | 0 | 0 | 0 | 0 | 0 | 0 | 0 |
| S_9_ | 0 | 0 | 0 | 0 | 0 | 0 | 0 | 0 | 1 | 0 | 0 | 0 | 0 | 0 | 0 | 0 | 0 | 0 | 0 | 0 | 0 | 0 |
| S_10_ | 0 | 1 | 1 | 1 | 1 | 0 | 1 | 0 | 1 | 1 | 1 | 0 | 0 | 0 | 0 | 0 | 0 | 0 | 0 | 0 | 0 | 0 |
| S_11_ | 0 | 0 | 0 | 0 | 0 | 0 | 0 | 0 | 0 | 0 | 1 | 0 | 0 | 0 | 0 | 0 | 0 | 0 | 0 | 0 | 0 | 0 |
| S_12_ | 1 | 1 | 1 | 1 | 1 | 0 | 1 | 1 | 1 | 1 | 1 | 1 | 1 | 0 | 0 | 1 | 0 | 0 | 0 | 0 | 0 | 0 |
| S_13_ | 1 | 1 | 1 | 1 | 1 | 0 | 1 | 1 | 1 | 1 | 1 | 1 | 1 | 0 | 0 | 1 | 0 | 0 | 0 | 0 | 0 | 0 |
| S_14_ | 0 | 0 | 0 | 0 | 0 | 0 | 1 | 1 | 0 | 0 | 0 | 0 | 0 | 1 | 0 | 0 | 0 | 0 | 0 | 0 | 0 | 0 |
| S_15_ | 1 | 0 | 0 | 0 | 0 | 0 | 0 | 0 | 1 | 0 | 1 | 0 | 0 | 0 | 1 | 0 | 0 | 0 | 0 | 0 | 0 | 0 |
| S_16_ | 1 | 1 | 1 | 1 | 1 | 0 | 1 | 1 | 1 | 1 | 1 | 1 | 1 | 0 | 0 | 1 | 0 | 0 | 0 | 0 | 0 | 0 |
| S_17_ | 1 | 1 | 1 | 1 | 1 | 0 | 1 | 1 | 1 | 1 | 1 | 1 | 1 | 0 | 1 | 1 | 1 | 0 | 0 | 0 | 0 | 0 |
| S_18_ | 1 | 1 | 1 | 1 | 1 | 1 | 1 | 1 | 1 | 1 | 1 | 1 | 1 | 0 | 0 | 1 | 0 | 1 | 1 | 0 | 0 | 0 |
| S_19_ | 1 | 1 | 1 | 1 | 1 | 0 | 1 | 1 | 1 | 1 | 1 | 1 | 1 | 0 | 0 | 1 | 0 | 0 | 1 | 0 | 0 | 0 |
| S_20_ | 1 | 1 | 1 | 1 | 1 | 1 | 1 | 1 | 1 | 1 | 1 | 1 | 1 | 0 | 1 | 1 | 0 | 1 | 1 | 1 | 1 | 1 |
| S_21_ | 0 | 1 | 1 | 1 | 1 | 0 | 1 | 0 | 1 | 1 | 1 | 0 | 0 | 0 | 0 | 0 | 0 | 0 | 0 | 0 | 1 | 0 |
| S_22_ | 1 | 0 | 0 | 0 | 0 | 1 | 0 | 0 | 1 | 0 | 1 | 0 | 0 | 0 | 1 | 0 | 0 | 0 | 0 | 0 | 0 | 1 |
